# Methodology for estimating energy and water consumption patterns in university buildings: case study, Federal University of Roraima (UFRR)

**DOI:** 10.1016/j.heliyon.2021.e08642

**Published:** 2021-12-21

**Authors:** Alissandra Pessoa Almeida, Vitor Sousa, Cristina Matos Silva

**Affiliations:** a CERIS, Instituto Superior Técnico, University of Lisbon, Av. Rovisco Pais, 1049-001, Lisbon, Portugal; b Federal University of Roraima, Av. Cap. Ene Garcez, 69304-000, Boa Vista, Roraima, Brazil; c CERIS, Instituto Superior Técnico, University of Lisbon, Department of Civil Engineering, Architecture and Georesources, Instituto Superior Técnico, University of Lisbon, Av. Rovisco Pais, 1049-001, Lisbon, Portugal

**Keywords:** University building, Water and energy consumption, End use of water and energy

## Abstract

Knowing water and energy consumption patterns sets the baseline for understanding their drivers and assessing the performance of potential measures to increase efficiency and/or reliability. These patterns can vary substantially depending on the building characteristics, on the building users and use, on the cultural, social, economic, environmental context in which the building is located, among many other factors. This article presents a general methodological framework for characterizing water and energy consumption patterns in buildings based on the evaluation of the characteristics of the equipments and appliances, as well as the type of users and the activities developed in each type of room. This allows estimating water and energy use, by end use per square meter and by roomtype. The methodological framework proposed was applied to the buildings of the Paricarana Campus of Federal University of Roraima (UFRR), Brazil, providing one of the few examples in the literature reporting water and energy consumption in university buildings in tropical climates. Universities, in most cases, represent large water and energy consumers with distinctive consumption drivers and patterns which have received limited attention when compared to other types of buildings (e.g., residential). The findings have shown that teaching rooms and administration rooms are the main consumers, representing 48% and 49% of the institution's energy and water consumption, respectively. Air conditioning is the biggest energy consumption (63%), while personal use represents 72% of the total water consumption in a building. The toilets represent a large water consumption in a university building (46.40%). Comparing different building uses, the central library is the highest consumer, due to the longest operating time and the highest occupational density. The methodological proposal intends to be a useful tool to support managers and decision-makers to understand the dynamics of consumption and then propose effective practices to reduce water and energy uses, as well as providing reference data for comparison with other educational institutions.

## 1 Introduction

Assessing the performance of existing buildings is crucial to meet the needs and expectations of both users and managers (Sink et al., 1984). Moreover, it is important to identify opportunities to establish refurbishment interventions or adequate standards, since a large portion of resources consumption and waste production takes place in buildings (Bertone et al., 2018; Dörr et al., 2013). Energy and water consumption are two of the most important variables/parameters to be measured (Abdelalim et al., 2015; Batlle et al., 2020; Bonnet et al., 2002). It is important to know/measure the consumption pattern of both when evaluating building's performance and forecasting the viability of potential improvement measures. Yet, many times, this information is not available.

On energy and water management, universities can be compared to small cities because of their size, the number of users and diversity of activities developed (Alshuwaikhat and Abubakar, 2008; Ragazzi and Ghidini, 2017). Universities are considered to be major energy (Chung and Rhee, 2014) and water (Alshuwaikhat and Abubakar, 2008) consumers. The lack of adequate management of these consumptions has great socioeconomic impacts in the cities where universities are located, especially in smaller cities (Ragazzi and Ghidini, 2017). Considering the high consumption and its great variation, it becomes increasingly necessary to use strategies to reduce water and energy consumption (Antunes and Ghisi, 2020). Thus, planning a more efficient Campus is imperative (Barnes and Jerman, 2002; Chung and Rhee, 2014; Koester et al., 2006).

The number of universities is growing exponentially worldwide to satisfy the increasing demand from population growth and a higher proportion of individuals seeking a higher education degree. This expansion, in the last decades, has been greater in developing countries such as Brazil and India (Valero and Van Reenen, 2019). Brazil experienced a strong expansion of higher education, between 1995 and 2014, enrolments of students had an increase in the public higher education system in the order of 134.5% (Mancebo et al., 2015). The expansion of higher education in Brazil did not envisage the sustainability of consumption resources such as energy and water (Marinho et al., 2014).

In this context, it has become a matter of global concern for university managers and decision makers to implement sustainability measures, as a result of awareness of the impacts that universities have on the environment (Alshuwaikhat and Abubakar, 2008). Furthermore, these institutions must play a leading role towards sustainable development (Viebahn, 2002), not only through education and research in the topic, but also through practical example by implementing measures to improve the performance of their infrastructures (Bellia et al., 2018; Bertone et al., 2018; Marinho et al., 2014).

The present research presents a review of the methodologies used to estimate water and energy consumption in buildings and proposes a holistic methodological framework to guide the consumption estimation process. In particular, an approach is proposed to indirectly estimate the consumptions and end uses in the context of lack of measured data based on the evaluation of the area and type of room. Indicators of water and energy use, by end use per square meter and by room type are proposed as outputs of the approach. These indicators can be applied in other buildings to estimate water and energy consumption. For this purpose, the type of room (e.g. classrooms, auditoriums), their typical consumption elements (e.g. air conditioning, lighting, cleaning), the frequency of use of the equipment and activities are identified, as well as the physical and temporal correction factors of the buildings.

The framework is detailed for characterizing water and energy consumption patterns in university buildings through the application to a case study - the buildings of the Federal University of Roraima (UFRR), Paricarana Campus, located in the state of Roraima in Brazil. The reasons to choose this case study were: i) water and energy consumption in university buildings are higher than in other types of buildings; ii) the literature review demonstrates that there is a lack of characterization of the water and energy consumption patterns in university buildings, particularly in tropical climates, and limited understanding regarding the relationship between consumption and the typological characteristics of buildings, occupation, types of activities and services offered; iii) Brazil has numerous universities with multiple campuses spread throughout the country; and iv) the data on water energy consumption in Brazil is scarce when compared to other countries.

The results obtained in this study are beneficial for understanding the energy and water performance of universities located in tropical climate regions in Brazil, and help to understand the main characteristics of energy and water consumption of different university buildings. It is expected that the presentation of the main factors that influence the energy and water consumption of the buildings will support all those involved in the energy and water management projects of these institutions in formulating and optimizing the operating strategies. The approach presented can be applied to any type of building to estimate energy and water consumption and end uses.

This paper is organized as follows. Section 2 presents an overview and previous studies on energy and water consumption in buildings. Section 3 describes the methodological framework proposed. Section 4 presents the case study and section 5 details the application and presents the results of the application of the proposed methodological framework to estimate water and energy consumption patterns in the case study. Finally, section 6 discusses the results and section 7 presents the conclusions.

## 2 Literature review

### 2.1 Water and energy consumption in buildings

Water and energy consumption in buildings depends on various exogenous (e.g., climate) and endogenous (e.g., fixtures and appliances) factors (Ma et al., 2017). Also, the building user's behavior plays a relevant role, with socio, economic, cultural, and ethical aspects affecting the behavior (Masoso and Grobler, 2010). As such, it is common practice to differentiate between residential and non-residential buildings for the purpose of characterizing the consumption patterns, since the type of building is a good proxy for the building and users characterization. Residential buildings are sometimes split into single-family and multi-family, but the non-residential buildings encompass a wider range of categories (Mudgal et al., 2009): i) commercial and public; ii) industrial; and iii) agricultural. The commercial and public buildings are further divided into (Mudgal et al., 2009): i) offices, ii) hotels and restaurants; iii) shops and retail trade services; iv) public cultural; v) hospitals and clinics; vi) offices; vii) educational buildings; and viii) sport facilities. Other classifications exist for commercial and public buildings. For instance, the energy and water surveys the U.S Energy Information Administration (EIA) consider the following building types (Department, 2010): i) health care; ii) service iii) public order and safety; iv) public assembly; v) lodging; vi) office; vii) mercantile; viii) vacant; ix) warehouse and storage; x) education; xi) religious worship; xii) food sales; xiii) food service; and xiv) other. This categorization is used because the average water and energy consumption patterns tend to be substantially distinct between each type of buildings, despite the significant variability of the pattern of each specific building within each category.

The studies on the characterization of water and energy consumption patterns can be organized based on the focus: i) the intensity; ii) the temporal pattern; and iii) the end-use distribution. The former focuses on getting average consumption values per number of occupants, area or other building characteristics considered relevant (e.g., number of beds in a hotel). Usually, the studies on this topic are statistical reports, often from official public entities (e.g., NRCan, 2011). The temporal pattern studies deal with how the total water or energy consumption varies over time, providing information regarding peak demand and seasonal patterns relevant for the design of the water and electrical infrastructures (Anda et al., 2013; Blokker et al., 2017; Butler, 1991; Rathnayaka et al., 2017). End-use distribution studies provide insight into how water and energy are effectively used (AWWA, 2016; Barreto, 2008; Bonnet et al., 2002; DeOreo et al., 1996a; Froehlich et al., 2009; Hall et al., 1988; Lee et al., 2012; L. Li et al., 2017; Rathnayaka et al., 2017; Sousa et al., 2019; Thackray et al., 1978; Vieira et al., 2007). This sets the baseline for understanding the drivers for water and energy consumption and allow the identification and evaluation of the technical, financial, and/or environmental performance of potential efficiency improvement solutions (Marinho et al., 2019; Marinoski and Ghisi, 2008).

A more recent class of studies aims at modeling consumption in buildings. Comparatively, modeling the energy consumption in buildings has been a more proficient field of study (e.g., (Aydinalp et al., 2004; Dong et al., 2005; Ekici and Aksoy, 2009; González and Zamarreño, 2005; Westphal and Lamberts, 2004; White and Reichmuth, 1996; Yik et al., 2001; Zmeureanu, 2003), due, in part, to the existence of more and better data. This, combined with the development of novel statistical-based models from the artificial intelligence field (e.g., artificial neural networks, support vector machines), unlocked the possibility of exploring hidden patterns in the data. The availability of physical-based tools for simulating the thermal performance of buildings (e.g., Energy Plus, FLUENT) is another factor explaining the number of studies on this topic. In addition to the numerous studies on this topic, there are also several literature reviews providing an overview of what has been done to date (e.g., Bourdeau et al., 2019; Ferrando et al., 2020; Foucquier et al., 2013; W. Li et al., 2017; Li et al., 2020; Swan and Ugursal, 2009). Some studies can also be found on water consumption modeling (e.g., Bennett et al., 2013; Parker, 2019; Walker et al., 2015), but they are scarcer and not for a specific type of buildings.

The basis for researching water and energy consumption patterns is collecting data. Broadly, there are three main categories of methods to record water and energy consumption: i) direct; ii) semi-direct; and iii) indirect. Direct methods rely on meters to record water or energy consumption. Technological developments in the meters and communication solutions have enabled the shift from manual readings to automatic data recording, which provided data with increased accuracy and resolution. Still, the implementation of these novel meters at a building scale has been more on energy than on water networks. The difficulty and cost of installing meters for recording water end-uses in detail have promoted the development of semi-direct methods for measuring water consumption. Basically, this category of methods rely on identifying the specific pattern of each water end-use device from a refined continuous record of one or more physical property associated to the building water infrastrucutre (e.g., pressure, flow, vibration). These are mostly proprietary solutions, such as Identiflow, Flow trace analysis, or HydroSense. Identiflow and Flow trace analysis collect flow data and the HydroSense collect pressure transients data and then the signature of each fixture and appliance is mapped (DeOreo et al., 1996b; Froehlich et al., 2009; Morrison and Friedler, 2015). Identiflow and Flow trace analysis have been used successfully in several studies (DeOreo et al., 1996a; Willis et al., 2011, 2013). The HydroSense is in an experimental phase (Morrison and Friedler, 2015). These technologies do not distinguish between fixtures or appliances with similar water signatures and loose precision when several similar devices are used simultaneously (Morrison and Friedler, 2015). Finally, indirect methods rely in audits and/or surveys. This is the base for official studies, such as the US Energy Information Administration surveys (https://www.eia.gov/consumption/).

### 2.2 Consumption in university building

Most of the studies found in the literature deal with residential buildings. In fact, the literature survey conducted identified 9 studies on the consumption of water (Abdelalim et al., 2015; Bonnet et al., 2002; Marinho et al., 2019; Meireles et al., 2014, 2018; Wichowski et al., 2019; Zhou et al., 2013) and 17 on the energy consumption (Abdelalim et al., 2015; Amber et al., 2017; Bonnet et al., 2002; Escobedo et al., 2014; Hong et al., 2011; Khoshbakht et al., 2018; Wang, 2016; Ward et al., 2008; Zhou et al., 2013) in university buildings that used different methodologies.

To compare the consumption of different universities Zhou et al. (2013) applied questionnaires to higher education institutions in Guangdong (China) Based on the results, the average water and energy consumption was determined considering the disciplines, natures and levels of the institutions.

To analyze the consumption of water, natural gas, and electricity of buildings on a university campus in Canada, Abdelalim et al. (2015) presented a methodology based on the analysis of Sankey diagrams and bar charts. This approach aims to facilitate the identification of the most inefficient buildings.

Methodologies suggesting the creation of consumption indicators were presented by Bonnet et al. (2002), Escobedo et al. (2014) and Batlle et al. (2020). Bonnet et al. (2002) presented a tool that allows addressing the diversity of activities and-end uses of water and energy at the University of Bordeaux. The method is based on the evaluation of the proportions of the surface areas of each activity, proposing reference values that can be applied in other buildings to estimate the consumption of water and energy. Escobedo et al. (2014) used data collected in energy audits carried out on buildings of the National Autonomous University of Mexico (UNAM) to present energy use indicators, by end use per square meter and by category of construction. Batlle et al. (2020) present a statistical modeling based on ISO 50001:2011 and ISO 50006:2014 to establish baselines and energy performance indicators in university buildings. A wide range of factors that influence energy consumption was considered in the model, as the types of activities carried out on the building, weather conditions, construction materials, air conditioning systems and occupation.

Statistical analyses to predict consumption in buildings were performed by Amber et al. (2017), who presented a mathematical equation to predict the daily use of electricity in university buildings, using multiple linear regression techniques. The results demonstrate that three variables, ambient temperature, number of working days and type of construction, influence the energy consumption of the buildings.

Khoshbakht et al. (2018) analyzed the energy consumption and energy use intensity of 80 university campus buildings in Australia. Consumption was related to the type of room and occupation conditions. The benchmarking technique used was stochastic frontier analysis. Reference values were presented for different activities and disciplines.

Although different authors have proposed methodologies to estimate consumption indicators for university buildings, the methodology presented in this article has some advantages over the others.

This approach refines the calculation of the consumption of university buildings, estimating them based on the proportions of each type of rooms that make up the buildings, being more comprehensive than the estimates that consider only the main characteristics of the buildings, made by Amber et al. (2017), Zhou et al. (2013) and Abdelalim et al. (2015). It was also considered in the calculations the proportion of the same type of room of all buildings present on the campus, replacing the use of “single activity” (or dominant activity) buildings for the estimation of the indicators, as done by Bonnet at al. (2002). This choice increases the sample size and reduces the standard deviations. The ease of application of the methodology is also emphasized, since it uses average values for the formulation of the indicators instead of statistical data as done by Batlle et al. (2020) and Khoshbakht et al. (2018). The approach also has the characteristic of being able to be applied in different realities, considering in the calculations correction factors that adapt the methodology to different situations.

## 3 Methodological framework

Estimating the performance and viability of the majority of water and energy efficiency measures requires prior knowledge and understanding of the consumption patterns with a level of resolution that most often is not available. Additionally, the nature and amount of data available is extremely variable, ranging from yearly bulk consumption approximations to high resolution (15 min intervals or less) water and energy end-uses (e.g., lighting, heating) data.

**Figure 1:**
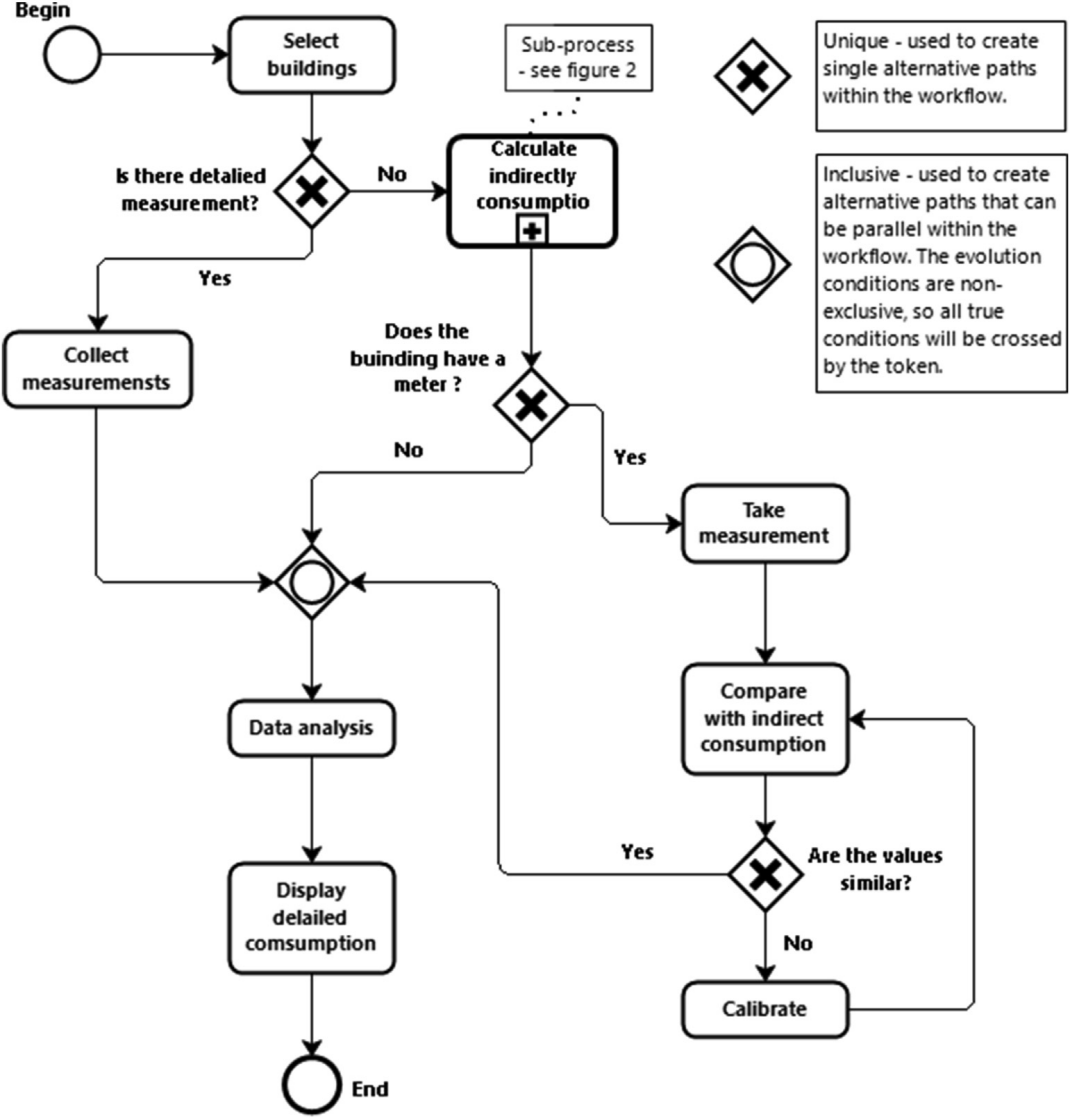
Methodological framework for estimating building water/energy consumption.

Within this context, the holistic methodological framework to guide the process of collecting water and energy data in buildings presented in Figure 1 is proposed.

The methodological framework considers three situations in terms of water and energy measurements availability: i) direct; ii) indirect; and iii) mixed.

Direct consumption metering is used when high-resolution automatic metering equipment is present in the building (e.g., smart meters), making it possible to identify the consumptions by end-uses. In this case, the methodology consists of selecting the buildings, collecting the consumption data, making the data analysis and presenting the results.

**Figure 2:**
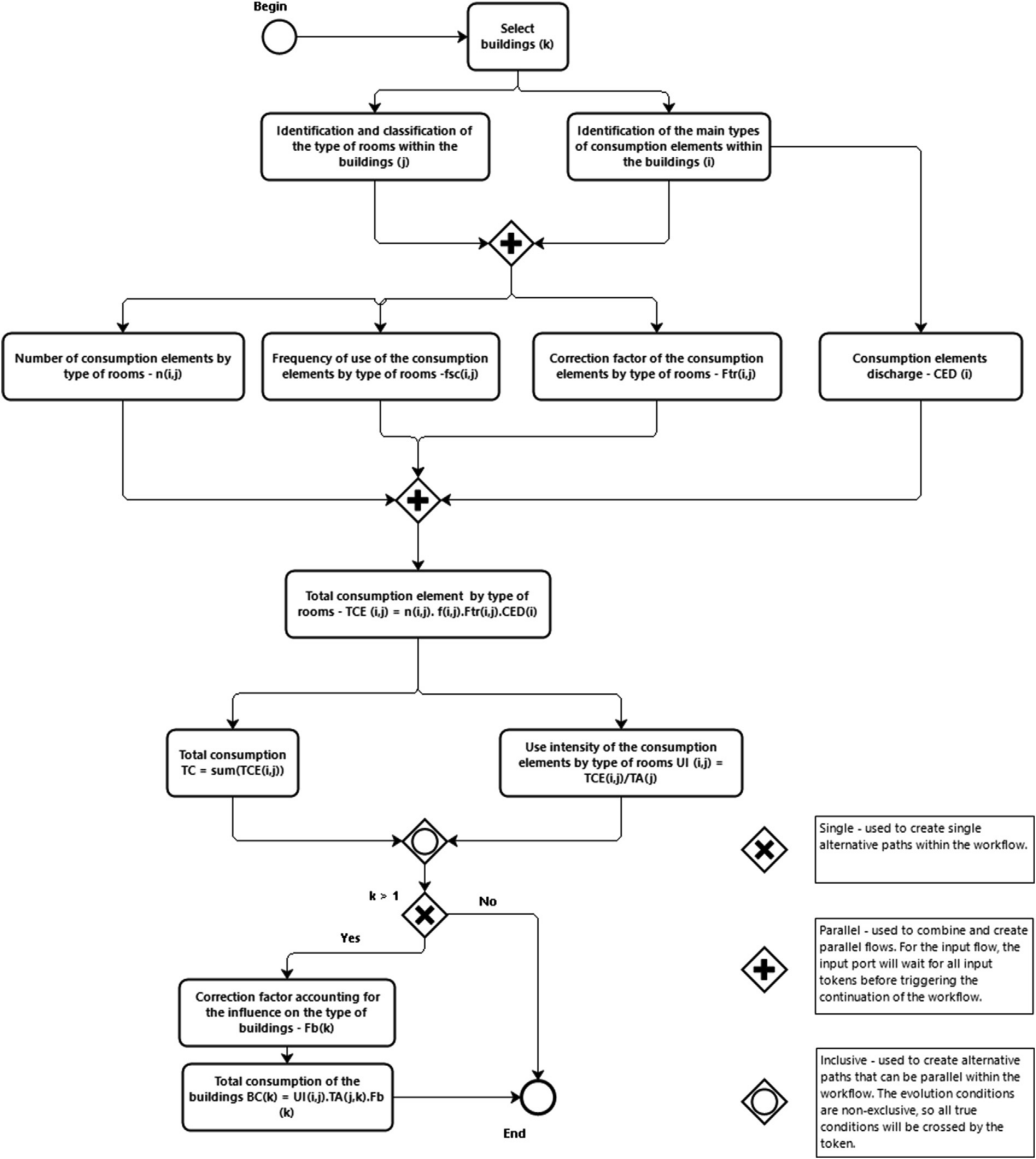
Methodology for estimating consumption indirectly in buildings.

When there is no metering equipment in buildings, it is necessary to obtain these values indirectly. This requires estimating consumption based on the characteristics of the building and the existing equipment as well as the services and uses in it, resorting to audits, questionnaires and/or direct observation, amongst other approaches. The indirect consumption estimate (Figure 2) is a sub-process of the general methodological framework shown in Figure 1, comprising the following steps: i) selection of the buildings; ii) identification and classification of the type of rooms within the building (e.g., teaching room, bathroom, office); iii) identification of the main types of consumption elements (CE) within the buildings (e.g., fixture; appliance; equipment; personal use devices and activities); iv) determination of the CE discharge (CED); v) quantification of the number (n) of CE by type of room; vi) estimation of the frequency (f) of use of the CE by classerooms; vii) identification and quantification of the correction factor (Ftr) accounting for the influence of the type of room on the CE consumption; viii) calculation of the total consumption element (TCE) by type of room; ix) calculation of the total energy (TEC) and water (TWC) consumption; x) determination of consumption intensity (CI) by type of room; xi) identification and quantification correction factor (Fb) accounting for the influence of the type of buildings; xii) estimation of the total energy (BEC) and water (BWC) consumption of the buildings.

The total consumption of each type of CE by type of room (TCE) is represented by the product of the consumption element discharge (CED), frequency of use of the CE (f), the number of CE (n), and the factor (Ftr) accounting for the influence of the type of room on the CE (equation 1). Total consumption (TC) is the sum of TCE (equation 2).

(1) $$\mathrm{TC}E_{\left(i,j\right)}=\mathrm{CE}D_{\left(i\right)}.n_{\left(i,j\right)}.f_{\left(i,j\right)}.\mathrm{Ft}r_{\left(i,j\right)}$$

(2) $$\mathrm{TC}=\overset{k}{\underset{i=1}{\sum }}\mathrm{TC}E_{\left(i,j\right)}$$

where, i is the type of the CE; and j is the type of room.

To determine consumption in each building, indicators of use intensity (UI) were used, represented as the ratio between TCE and the total area (TA) each type of room (equation 3).

(3) $$UI_{\left(i,j\right)}=\frac{\mathrm{TC}E_{\left(i,j\right)}}{TA_{\left(j\right)}}$$

The building consumption (BC) is represented as the sum of the product of UI by the total area (TA) each type of room by the factor (Fb) accounting for the influence on the type of buildings (equation 4).

(4) $$BC_{k}=\overset{n}{\underset{i=1}{\sum }}\overset{m}{\underset{j=i}{\sum }}UI_{\left(i,j\right)}.TA_{\left(j\right)}.Fb_{\left(k\right)}$$

In many countries, the most common situation is the existence of manual reading meters providing aggregate water and energy consumption records. This solution is frequently present when utilities are payed based on consumption, resulting, usually, in data at a monthly time scale with a building or group of buildings resolution. Under these conditions, it is possible to adopt a mixed consumption measurement approach, consisting in measuring the detailed consumption indirectly (as described above) and comparing the estimates against the total consumption recorded by the meters. This enables calibrating the bulk indirect consumption estimates using measured data.

## 4 Case study

### 4.1 Presentation

The case study selected was the Paricarana Campus of the Federal University of Roraima (UFRR). With approximately 10 000 students enrolled, it is the largest university campus in the state of Roraima. It is located in the central region of Boa Vista, capital of Roraima, on the banks of the Rio Branco in the legal Amazon, Brazil. The climate in Boa Vista is considered humid tropical (Aw type according to Köppen), with dry winter and rainy summer. Fall and spring are hardly noticed and, according to the National Institute of Meteorology (INMET), the temperature ranges from 22.3 °C to 33.7 °C, with an annual average of 27.4 °C, and there are 1 420 h of sunlight per year. The annual average relative humidity is of 74.9% and the average annual precipitation is over 1 400 mm, concentrated between the months of May to August.

The campus occupies an area of approximately 840 000 m^2^ and incorporates 79 buildings/complexes totaling a building area of over 87 000 m^2^. The university lacks any detailed records of water and energy consumption to allow assessing the performance of potential improvement measures. The data is available at a monthly scale and by groups of buildings, since the meters available cover zones of the campus and not each building individually. This corresponds to a mixed situation defined in the methodological framework.

**Figure 3:**
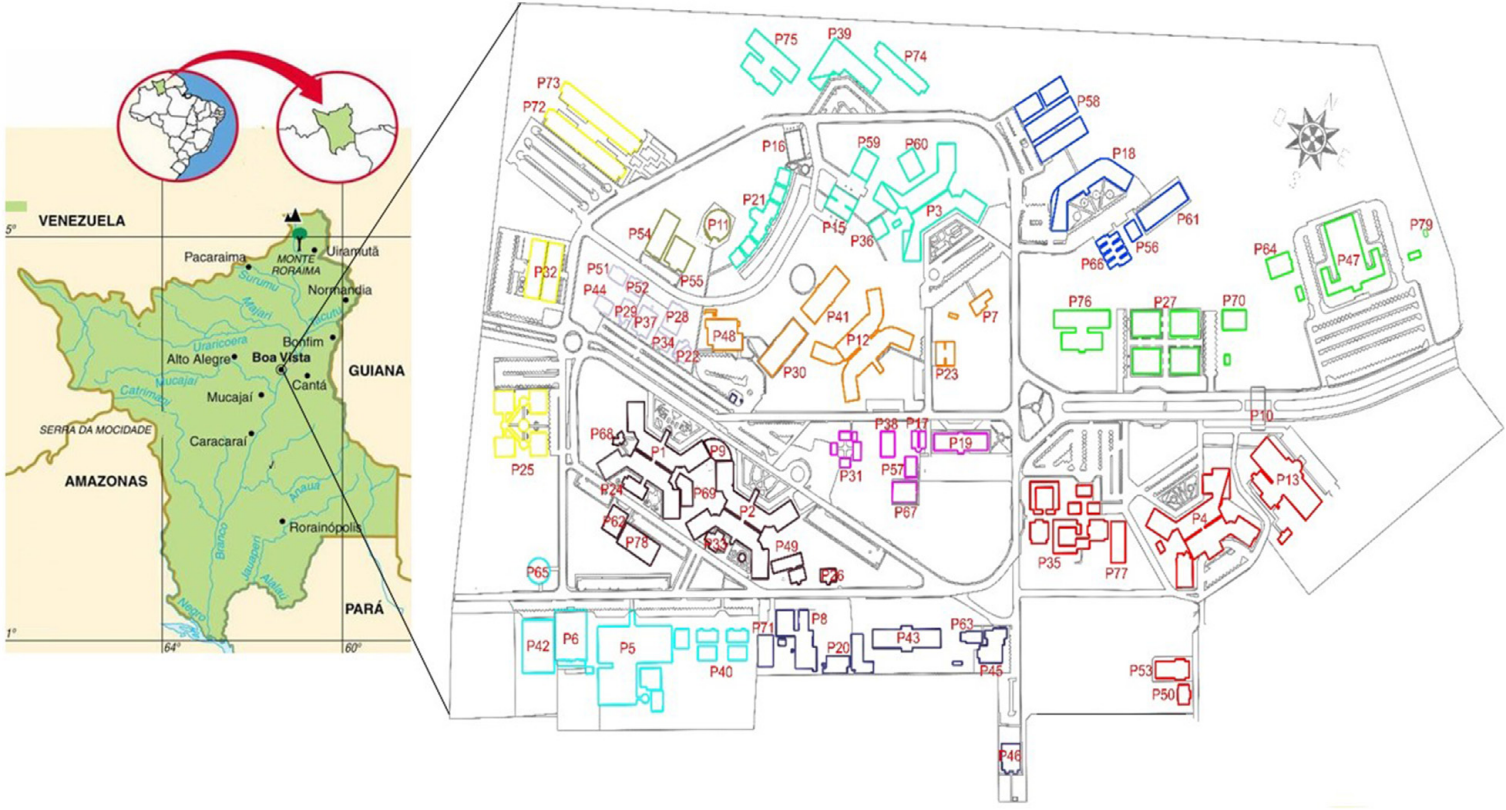
Paricarana Campus buildings.

To deal with the UFRR campus complexity, the buildings were grouped based on the availability of water and energy consumption records. The buildings connected to the same meter comprised a group, excluding the empty and inactive buildings. A plan view of the campus is presented in Figure 3, with the buildings identified by codes and colors dividing them by water consumption zones.

The buildings in each group were then characterized in terms of the drivers for water and energy consumption, namely the: i) occupancy; ii) use; and iii) equipment.

The occupancy comprises the number and the class of individuals using the buildings. The university population was classified into: i) students; ii) academic staff, and; iii) administrative staff. The student population was represented as the sum of high school, undergraduate and postgraduate students.

The use was categorized taking into account the spatial organization of the buildings and the activities developed in each room. The following uses were considered: i) administrative areas (AA); ii) teaching areas (TA); iii) library areas (LIA); iv) laboratory areas (LA); v) office areas (OA); vi) food preparation areas (FPA); vii) bathrooms areas (BA); viii) support room areas (SA); and ix) other areas (OTA).

The equipment or services available in each room were characterized based on their water and energy consumption characteristics. The equipment was split into appliances for: i) personal use (water - toilets, urinal, washbasin taps, showers, and drinking fountains; energy - computers, cell phone/tablet and projectors); and ii) general use (water - cleaning operations; energy - air conditioning and lighting). This categorization provides an organizational framework to collect the data on the existing water and energy consuming equipment, as well as their use pattern.

### 4.2 Data collection

The data collection required using various approaches, including: i) gathering historical records; ii) buildings surveys; iii) measurements on end-use equipment; iv) interviews with building managers; and v) surveys to the population and services.

The historical data available present information on the: i) bulk water and energy consumption in the campus; and ii) features of the building that may affect water and energy consumption.

The potable water of the Paricarana campus comes from 3 artesian wells located within the campus perimeter. The water is pumped to two elevated reservoirs, with 30 m^3^ and 20 m^3^, before being distributed through the various buildings. The campus water distribution network is comprised of a closed main ring from where derivations supply groups of buildings, defining consumption zones equipped with water meters. The consumption recorded on the water meters is not monitored and does not account for all water consumed, so the total water consumption was estimated from the hydraulic characteristics of the pumps in the wells and the respective operating time throughout the year. The Paricarana campus has a single electricity meter with monthly records. For this research, it was possible to collect the records from 2015 to 2017.

The features of the buildings that were investigated include: i) the physical characteristics of the building; ii) the areas of the buildings; iii) the activities developed in the rooms; iv) the occupation of the buildings; and v) the number and characteristic of energy (e.g., illumination, cooling) and water (e.g., bathroom fixtures) consuming devices and equipment.

The physical characteristics were obtained from the engineering and architecture designs and focused on collecting information about the envelope (e.g., wall, windows, roofs composition) and the university layout (e.g., location of classrooms, bathrooms, offices). The occupancy of the buildings was obtained from the Human Resources Directorate (DDRH), UFRR management report of 2017 (UFRR, 2018) and UFRR institutional websites. The methodology used to estimate the population of undergraduate students was proposed by Brazil's Tribunal de Contas da União (TCU) (TCU, 2002), considering for the calculation the number of full-time students (AGTI). The DDRH report was used to quantify the number of administrative and academic staff of the Campus.

The number of luminaires and air conditioning devices in the rooms was obtained by analyzing the electrical projects and reports of different buildings. Regarding lighting, the design criteria followed the ABNT (2004), requiring a point of light for the first 6m^2^ and one point for every additional 4m^2^. For air conditioning, the criteria used was 1 000 Btus per m^2^ of the room areas (university defined criteria).

The buildings’ surveys were conducted to check the accuracy of the historical records, namely the physical characteristics of the buildings and the number and performance of the energy and water consuming equipment. The latter were complemented with on-situ measurement, particularly the flow rates of the taps, washbasins and showers since their effective discharge depends on the water pressure. The discharges were determined using the volume-time measurement approach, recording the time to fill 1 L. Three measurements were done for each fixture and the reported flow rate corresponds to the average value.

The interviews with the administrators of the buildings and of the university allowed to identify the nature of the activities carried out in the rooms and buildings. This enabled estimating the operating time of lighting and cooling equipment. It also allowed a broad view of the consumer activities in the buildings.

In addition to the interviews with the administrators, the estimation of the consumption habits of the academic population was done resorting to indirect surveys. A questionnaire was sent via email to the entire community during June 2017, ensuring anonymity of the replies and attempting to obtain the most complete portrait of the real behavior of the population. The questionnaire consisted of 3 parts: i) definition of the user profile, with questions related to the link to the university, age, gender and length of stay; ii) definition of water consumption pattern, inquiring the frequency of activities such as drinking water, toilet and urinal flushing, hands washing, teeth brushing and bathing; and iii) definition of electricity consumption pattern, inquiring about the use of electronic equipment during their stay at UFRR. After the initial tests with the questionnaire format, it was found that few individuals replied to questions regarding the time of water use, therefore it was necessary to resort to reference values (e.g. Barreto, 2008; DOCOL, 2019) to estimate the average water consumed in each consumption activity.

The water consumption for cleaning the rooms was determined by surveying directly the service. In these surveys, the volume of water consumed per square meter of clean area for the washing the floor and the frequency of the cleaning operations were recorded.

The data used in the research was collected with permission from the university's top management, namely the Rector and the Infrastructure Manager, complying with the ethical standards of the institution. The interviews with the building managers and the surveys to the installations and services were conducted by the first author, also a member of the university technical staff in leave to develop a PhD at IST. Voluntary participation questionnaires were distributed to the academic community, whose anonymity was ensured at all times. The release of the questionnaires was accompanied by a full disclosure that the data collected would be used for scientific research.

### 4.3 Consumption estimation

The estimation of the water and energy consumption was detailed only for the most significant end-uses. The accuracy of the data collection approaches used, along with the simplifications that were required to assume, imply a degree of uncertainty that renders the smaller end-uses estimates meaningless.

Regarding energy consumption, it was found that the most representative portions of consumption in the UFRR buildings were those related to personal use, cooling and lighting. Total energy consumption was divided into these three components: i) lighting (ECL); ii) air conditioning (ECAC); and iii) electronic devices (ECE).

The most significant water end-uses considered were: i) personal use (CWP); and ii) cleaning (CWC). The water for personal use include drinking water, sanitation services and personal hygiene. The water used in cleaning activities only accounts for internal cleaning operations in the various building. Water consumption for food preparation and academic activities may also represent a significant share, but the information available was not enough to detail adequately these end-uses.

The water and energy end-uses were detailed in terms of: i) population consumption (CEE and CWP); ii) equipment consumption (CEL and CEAC); and iii) activities consumption (CWC).

## 5 Application and results

### 5.1 Campus characterization

The total campus population was split in: i) students (4 022); ii) administrative staff (563); and iii) academic staff (611). The students are the largest group, representing 77% of the total, followed by the academic staff (12%) and administrative staff (13%). The number of occupants represents an important driver of the energy and water consumption in the buildings (Abdelalim et al., 2015; Cheng and Hong, 2004; Raatikainen et al., 2016). Occupancy in public buildings is a complicated issue, since it is never known exactly how many users will access the building on a daily basis, and it is only possible to estimate the number (Cheng and Hong, 2004). The occupancy patterns differ significantly with the type of room, but these patterns are unknown. Given the absence of measurable data, the population in each type of room was calculated using engineering methods. The total population of the buildings was distributed by the rooms class considering the administrative staff in the administrative rooms, the academic staff in the offices, and the students in the teaching rooms, laboratories, libraries and support rooms.

**Figure 4:**
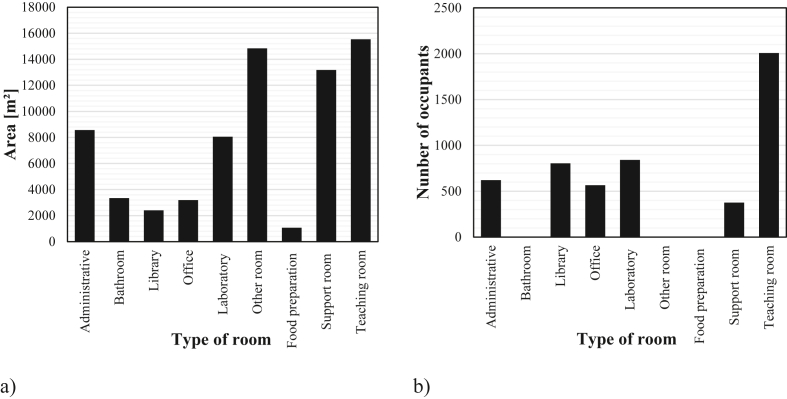
Distribution of the areas (a) and occupation (b) of the type of room of buildings.

The type of room with the largest areas and occupancy at the Campus are the teaching rooms. Rooms such as circulation and warehouses and the support areas, that contain gymnasiums and auditoriums, represent large areas of the Campus, however they have a very low occupation. It was observed that the administrative areas are twice that of office, even though there are more academic staff than administrative staff. Considering the built area and the population, libraries are the room with the highest occupancy rate. The bathrooms, other rooms and food preparation do not have a fixed population, since they are environments that only serve as support for the activities developed in the buildings (Figure 4).

The occupancy of the building rooms was estimated considering that it corresponds to the ratio between the population of the room by the total area of the rooms.

**Figure 5:**
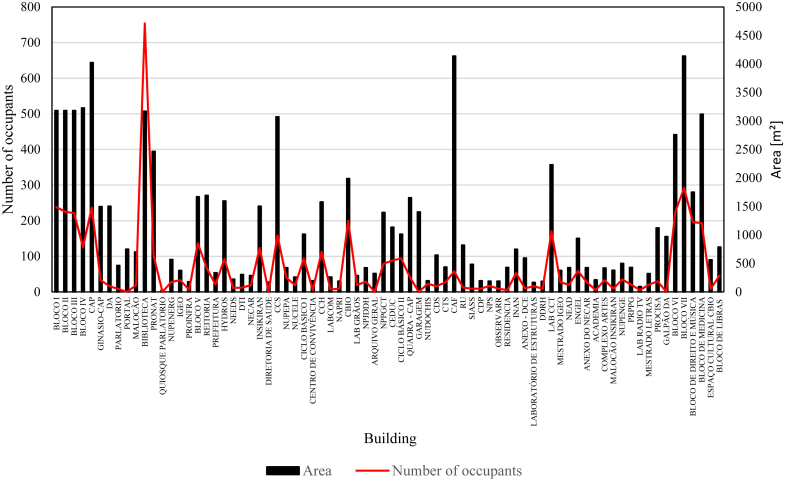
Area and occupation of buildings on the Paricarana campus.

Figure 5 shows the number of occupants and the area of each of the campus buildings. In general, buildings with larger areas have the largest populations. The values do not represent the instantaneous occupancy of the buildings, but rather daily averages. Eventual weekly and monthly variations due to the schedules (e.g., more classes in some days) or calendar (e.g., classes, examinations, holidays) patterns were not accounted for.

### 5.2 Indirect consumption

Since the buildings do not have a direct measurement system, the consumption of the buildings was calculated indirectly. The first step was to register the consumption of the CE present in the buildings, in terms of: i) equipment consumption (EC); ii) population consumption (PC) and; and iii) activities consumption (AC). Table 1 presents a summary with the code of the CE (equipment, device or activities) and their respective consumption.

**Table 1:** CE consumption.

Code	Equipment/Device/Activity	consumption	Unit
EC1	Air conditioning 12000 BTUS	763	kWh/1000h
EC2	Air conditioning 18000 BTUS	1160	kWh/1000h
EC3	Air conditioning 24000 BTUS	1623	kWh/1000h
EC4	Air conditioning 30000 BTUS	1820	kWh/1000h
EC5	Air conditioning 48000 BTUS	3223	kWh/1000h
EC6	Air conditioning 60000 BTUS	3896	kWh/1000h
EC7	Lighting (2 × 16w)	40	kWh/1000h
EC8	Lighting (2 × 32w)	78	kWh/1000h
PC1	Computer	180	kWh/1000h
PC2	Laptop	90	kWh/1000h
PC3	Projector	300	kWh/1000h
PC4	Mobile phone/tablet chargers	5	kWh/1000h
PC5	Drink	0.25	l/use
PC6	Toilet	6	l/use
PC7	Urinal	1.2	l/use
PC8	Tap (wash hands)	1.38	l/use
PC9	Tap (wash teeth)	1.38	l/use
PC10	Shower	35.3	l/use
AC1	Damp cloth in the floor	0.32	l/m^2^
AC2	Wash the floor	1.23	l/m^2^

Next, the CE present in the rooms of the Campus buildings were quantified and classified: i) air conditioning and lighting equipment were counted; ii) the personal use devices were estimated based on the number of users in the room; and iii) the area cleaned was used for quantifying the cleaning activities (Table 2).

**Table 2:** Number of CE in the rooms.

Code	AA	BA	LIA	OA	LA	OA	FPA	SA	TA
EC1	87	0	0	33	29	0	35	14	3
EC2	99	0	4	141	19	0	12	16	15
EC3	43	0	1	3	17	0	9	15	60
EC4	92	0	2	16	71	0	6	19	43
EC5	34	0	1	7	65	0	2	41	224
EC6	19	0	42	0	38	0	4	99	46
EC7	0	671	0	0	0	1	0	0	0
EC8	1814	0	589	643	1813	3625	226	3169	3524
PC1	611	0	803	563	842	0	0	374	2003
PC2	611	0	803	563	842	0	0	374	2003
PC3	611	0	803	563	842	0	0	374	2003
PC4	611	0	803	563	842	0	0	374	2003
PC5	611	0	803	563	842	0	0	374	2003
PC6	611	0	803	563	842	0	0	374	2003
PC7	611	0	803	563	842	0	0	374	2003
PC8	611	0	803	563	842	0	0	374	2003
PC9	611	0	803	563	842	0	0	374	2003
PC10	611	0	803	563	842	0	0	374	2003
AC1	8421	3599	2409	3177	8417	14513	1517	12467	15716
AC2	8421	3599	2409	3177	8417	14513	1517	12467	15716

The frequency of use of the equipment was estimated considering the time in which the rooms are used. The frequency of use of devices for personal use was obtained from the 360 responses to the inquiries done to students, academic staff and administrative staff. In Table 3, the representation of the sample of respondents is verified against the respective population for a confidence level of 95%. The student population presented the smallest error 6%. For academic staff and administrative staff the errors were higher, but less than 15% in any case.

**Table 3:** Enquiries respondents characteristics.

Type	Population	Replies	Response rate	Sample error
Students	4400	251	5.70%	6.00%
Teacher	562	46	8.19%	14.00%
Administrative staff	661	63	9.53%	12.00%
Total	5623	360	6.40%	5.00%

The sample is balanced regarding gender division, with an average of 57.6% female and 42.4% male. Most students are under 35, and academic staff and administrative staff are between 25 and 50. With regard to the length of stay at the university, there are approximate values for all population categories with an average of 7.73 h per working day (Table 4).

**Table 4:** Gender and demographic distribution of the respondents.

Type	Gender		Age (years)					Length of stay (h/day)
	Female	Male	17–24	25–35	36–50	51–60	>60	
Students	58.17%	41.83%	74.90%	17.13%	7.17%	0.40%	0.40%	7.51
Teacher	52.17%	47.83%	0.00%	32.61%	30.43%	30.43%	6.52%	8.05
Administrative staff	58.73%	41.27%	1.59%	53.97%	33.33%	9.52%	1.59%	8.35
Total	57.50%	42.50%	52.50%	25.56%	14.72%	5.83%	1.39%	7.73

The rooms are cleaned every day, Monday to Friday, with a damp cloth with detergent. On Saturdays, the rooms are washed with water and washing powder. In the wet areas (bathrooms, pantry and kitchen), cleaning is done daily by washing the floor. The laboratories are cleaned every day from Monday to Friday, with the damp cloth with detergent.

Since the frequencies are strongly influenced by the academic calendar and the days of the week, it was decided to work with different frequencies (Table 5) for: i) class days; ii) Saturdays; and iii) class breaks. On Sundays and public holidays, the buildings were considered closed, without consumption.

**Table 5:** Use frequency of the CE on school days, Saturdays and class breaks.

Cod	AA	BA	LIA	OA	LA	OTA	FPA	SA	TA
**School days**									
EC1	8.00	0.00	12.00	6.00	6.00	0.00	8.00	4.00	8.00
EC2	8.00	0.00	12.00	6.00	6.00	0.00	8.00	4.00	8.00
EC3	8.00	0.00	12.00	6.00	6.00	0.00	8.00	4.00	8.00
EC4	8.00	0.00	12.00	6.00	6.00	0.00	8.00	4.00	8.00
EC5	8.00	0.00	12.00	6.00	6.00	0.00	8.00	4.00	8.00
EC6	8.00	0.00	12.00	6.00	6.00	0.00	8.00	4.00	8.00
EC7	8.00	4.00	12.00	6.00	4.00	4.00	8.00	4.00	8.00
EC8	8.00	4.00	12.00	6.00	4.00	4.00	8.00	4.00	8.00
PC1	6.13	0.00	1.04	2.65	1.04	0.00	0.00	1.04	6.13
PC2	0.72	0.00	1.97	0.36	1.97	0.00	0.00	1.97	0.72
PC3	0.00	0.00	0.18	0.78	0.18	0.00	0.00	0.18	0.00
PC4	0.88	0.00	1.93	0.45	1.93	0.00	0.00	1.93	0.88
PC5	4.95	0.00	4.50	5.00	4.50	0.00	0.00	4.50	4.95
PC6	2.98	0.00	2.55	2.72	2.55	0.00	0.00	2.55	2.98
PC7	0.67	0.00	0.68	0.83	0.68	0.00	0.00	0.68	0.67
PC8	4.52	0.00	3.50	3.91	3.50	0.00	0.00	3.50	4.52
PC9	0.49	0.00	0.72	0.42	0.72	0.00	0.00	0.72	0.49
PC10	0.09	0.00	0.09	0.02	0.09	0.00	0.00	0.09	0.09
AC1	1.00	0.00	1.00	1.00	1.00	1.00	1.00	1.00	1.00
AC2	0.00	1.00	0.00	0.00	0.00	0.00	0.00	0.00	0.00
**Saturdays**									
EC1	0.00	0.00	4.00	0.00	0.00	0.00	0.00	0.00	4.00
EC2	0.00	0.00	4.00	0.00	0.00	0.00	0.00	0.00	4.00
EC3	0.00	0.00	4.00	0.00	0.00	0.00	0.00	0.00	4.00
EC4	0.00	0.00	4.00	0.00	0.00	0.00	0.00	0.00	4.00
EC5	0.00	0.00	4.00	0.00	0.00	0.00	0.00	0.00	4.00
EC6	0.00	0.00	4.00	0.00	0.00	0.00	0.00	0.00	4.00
EC7	0.00	0.00	4.00	0.00	0.00	0.00	0.00	0.00	4.00
EC8	0.00	0.00	4.00	0.00	0.00	0.00	0.00	0.00	4.00
PC1	0.00	0.00	0.35	0.88	0.35	0.00	0.00	0.35	0.35
PC2	0.00	0.00	0.66	0.12	0.66	0.00	0.00	0.66	0.66
PC3	0.00	0.00	0.06	0.26	0.06	0.00	0.00	0.06	0.06
PC4	0.00	0.00	0.64	0.15	0.64	0.00	0.00	0.64	0.64
PC5	0.00	0.00	1.50	1.67	1.50	0.00	0.00	1.50	1.50
PC6	0.00	0.00	0.85	0.91	0.85	0.00	0.00	0.85	0.85
PC7	0.00	0.00	0.23	0.28	0.23	0.00	0.00	0.23	0.23
PC8	0.00	0.00	1.17	1.30	1.17	0.00	0.00	1.17	1.17
PC9	0.00	0.00	0.24	0.14	0.24	0.00	0.00	0.24	0.24
PC10	0.00	0.00	0.03	0.01	0.03	0.00	0.00	0.03	0.03
AC1	0.00	0.00	0.00	0.00	0.00	0.00	0.00	0.00	0.00
AC2	1.00	1.00	1.00	1.00	0.00	1.00	1.00	1.00	1.00
**Class break**									
EC1	8.00	0.00	12.00	0.00	6.00	0.00	0.00	0.00	0.00
EC2	8.00	0.00	12.00	0.00	6.00	0.00	0.00	0.00	0.00
EC3	8.00	0.00	12.00	0.00	6.00	0.00	0.00	0.00	0.00
EC4	8.00	0.00	12.00	0.00	6.00	0.00	0.00	0.00	0.00
EC5	8.00	0.00	12.00	0.00	6.00	0.00	0.00	0.00	0.00
EC6	8.00	0.00	12.00	0.00	6.00	0.00	0.00	0.00	0.00
EC7	8.00	0.00	12.00	0.00	4.00	4.00	0.00	0.00	0.00
EC8	8.00	0.00	12.00	0.00	4.00	4.00	0.00	0.00	0.00
PC1	6.13	0.00	0.00	2.65	0.00	0.00	0.00	0.00	1.04
PC2	0.72	0.00	0.00	0.36	0.00	0.00	0.00	0.00	1.97
PC3	0.00	0.00	0.00	0.78	0.00	0.00	0.00	0.00	0.18
PC4	0.88	0.00	0.00	0.45	0.00	0.00	0.00	0.00	1.93
PC5	4.95	0.00	0.00	5.00	0.00	0.00	0.00	0.00	4.50
PC6	2.98	0.00	0.00	2.72	0.00	0.00	0.00	0.00	2.55
PC7	0.67	0.00	0.00	0.83	0.00	0.00	0.00	0.00	0.68
PC8	4.52	0.00	0.00	3.91	0.00	0.00	0.00	0.00	3.50
PC9	0.49	0.00	0.00	0.42	0.00	0.00	0.00	0.00	0.72
PC10	0.09	0.00	0.00	0.02	0.00	0.00	0.00	0.00	0.09
AC1	1.00	0.00	1.00	1.00	0.00	1.00	0.00	1.00	0.00
AC2	0.00	0.50	0.00	0.00	0.00	0.00	1.00	0.00	0.00

The Paricarana Campus is located in a region with relatively stable weather throughout the year (except for the difference between wet and dry seasons) and the buildings present very similar construction characteristics, at least in terms of energy and water efficiency. Considering these specificities, the correction factors used were related to the intensity of use of the rooms and occupancy of the buildings.

As the use of rooms varies with time. To adjust consumption to a specific period (the year of 2017), a factor of the intensity of use (FU) was used. This factor represents the relationship between the current usage time of the room (Hat) and the maximum usage time (Hmax) (Batlle et al., 2020). The factor was applied to correct the consumption of the equipment (EC) in the rooms.

The CE by rooms was calculated based on the year 2017, considering: i) 228 days of classes; ii) 40 days of class breaks; iii) 45 Saturdays; and iv) 52 Sundays and public holidays (Table 6). The total energy consumption $\text{TEC}=\overset{12}{\underset{i=1}{\sum }}\overset{9}{\underset{j=i}{\sum }}EC_{\left(i,j\right)}$ and water consumption $\text{TWC}=\overset{20}{\underset{i=13}{\sum }}\overset{9}{\underset{j=i}{\sum }}EC_{\left(i,j\right)}$ of the Campus are 5002.35 GWh e 46 786 m³ respectively.

**Table 6:** The CE by type of room of the Buildings of the Paricarana Campus.

Code	AA	BA	LIA	OA	LA	OTA	FPA	SA	TA
EC1	78276	0	0	32553	21950	0	7306	5845	1835
EC2	135419	0	12606	211459	21864	0	3809	10156	13948
EC3	82295	0	4409	6295	27370	0	3996	13322	78060
EC4	197445	0	9889	37648	128186	0	2988	18922	62733
EC5	129219	0	8756	29168	207819	0	1764	72309	578717
EC6	87289	0	444555	0	146864	0	4264	211057	143660
EC7	0	1369	0	0	0	12	0	0	0
EC8	166847	0	124815	64842	140283	84146	4823	135258	220337
PC1	180680	0	36528	76000	38302	0	0	17013	106114
PC2	10611	0	34596	5191	36277	0	0	16113	100502
PC3	0	0	10537	37283	11049	0	0	4908	30610
PC4	720	0	1883	358	1974	0	0	877	5470
PC5	203	0	220	199	230	0	0	102	638
PC6	2928	0	2985	2600	3130	0	0	1390	8673
PC7	132	0	159	159	167	0	0	74	463
PC8	1021	0	942	860	988	0	0	439	2738
PC9	111	0	194	92	203	0	0	90	563
PC10	543	0	634	135	664	0	0	295	1841
AC1	722	0	207	272	614	1245	111	1069	1147
AC2	466	1297	133	176	0	803	159	690	870

Considering the CE by type of room and the areas of the rooms of the buildings, the use intensity of the CE was determined. Energy use intensity of the CE was represented in kWh/m^2^/year and water use intensity of the CE in m³/m^2^/year (Table 7).

**Table 7:** Use intensity of the CE by type of rooms.

Code	AA	BA	LIA	OA	LA	OTA	FPA	SA	TA
EC1	9.14	0.00	0.00	10.20	2.73	0.00	6.84	0.44	0.12
EC2	15.81	0.00	5.23	66.24	2.71	0.00	3.57	0.77	0.90
EC3	9.61	0.00	1.83	1.97	3.40	0.00	3.74	1.01	5.02
EC4	23.05	0.00	4.11	11.79	15.92	0.00	2.80	1.44	4.04
EC5	15.08	0.00	3.63	9.14	25.80	0.00	1.65	5.49	37.25
EC6	10.19	0.00	184.54	0.00	18.24	0.00	3.99	16.01	9.25
EC7	0.00	0.41	0.00	0.00	0.00	0.00	0.00	0.00	0.00
EC8	19.48	0.00	51.81	20.31	17.42	5.67	4.52	10.26	14.18
PC1	21.09	0.00	15.16	23.81	4.54	0.00	0.00	1.29	6.83
PC2	1.24	0.00	14.36	1.63	4.30	0.00	0.00	1.22	6.47
PC3	0.00	0.00	4.37	11.68	1.31	0.00	0.00	0.37	1.97
PC4	0.08	0.00	0.78	0.11	0.23	0.00	0.00	0.07	0.35
PC5	0.02	0.00	0.09	0.06	0.03	0.00	0.00	0.01	0.04
PC6	0.34	0.00	1.24	0.81	0.37	0.00	0.00	0.11	0.56
PC7	0.02	0.00	0.07	0.05	0.02	0.00	0.00	0.01	0.03
PC8	0.12	0.00	0.39	0.27	0.12	0.00	0.00	0.03	0.18
PC9	0.01	0.00	0.08	0.03	0.02	0.00	0.00	0.01	0.04
PC10	0.06	0.00	0.26	0.04	0.08	0.00	0.00	0.02	0.12
AC1	0.08	0.00	0.09	0.09	0.08	0.08	0.10	0.08	0.07
AC2	0.05	0.39	0.06	0.06	0.00	0.05	0.15	0.05	0.06

**Figure 6:**
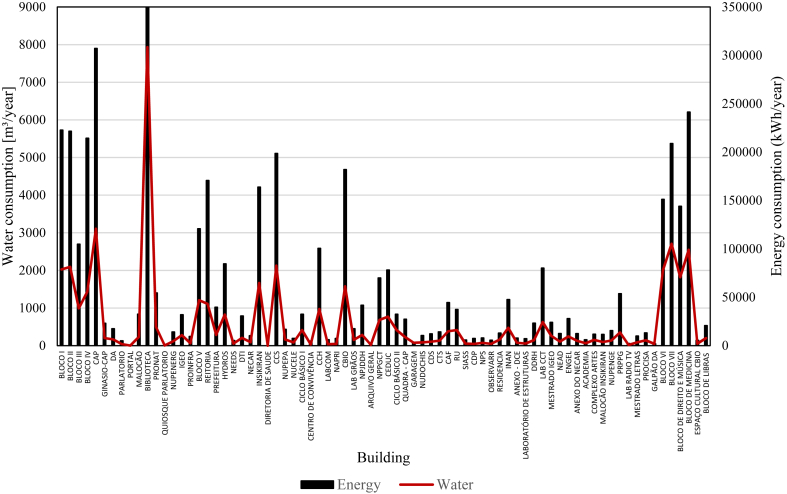
Water and energy consumption of the buildings.

As the occupancy of the buildings varies with time, to adjust consumption to a specific period (the year of 2017) a factor of the intensity of occupancy of the buildings (FO) was used. This factor represents the relationship between the current building population and the maximum building population. To calculate consumption this factor was determined considering three levels: i) low occupation, when the occupancy of the building is less than 50% of the total capacity, ii) average occupation, when the occupancy of the building up is between 50% and 75% of the total capacity; and iii) high occupation, when the occupancy of the building is higher than 75% of the total capacity. The values of FO were calibrated based on the available measurements considering the average occupancy as the reference (FO = 1 for both energy and water). For energy, the FO was approximated to 0.5 (low occupancy) and 1.47 (high occupancy). Similar values were approximated for water, with 0.5 (low occupancy) and 1.50 (high occupancy). The energy and water consumption of the campus buildings are presented in Figure 6.

### 5.3 Calibration

The measured energy consumption of the Paricarana Campus can be split into buildings and infrastructure. In infrastructure, the most representative consumptions are street lighting and water pumping. In 2017, the infrastructure energy consumption was 5 700 MWh, of which street lighting was 460 MWh and water pumping 35 MWh. The buildings represented 91% of all energy consumed on campus, totaling 5 205 MWh in 2017.

The total water consumption of the campus was obtained from the pump's operation. In 2017 it was 173 400.00 m³. This total value includes leakage and watering of gardens. Thus, the available sector measurements were used to estimate the consumption within the buildings. The water measurements on zones 1 and 10, which aggregate 11 buildings, recorded 4 389 m³ in 2017.

Comparing the consumption values obtained with the application of the methodology (calculated values) with the results of the measurements made on the meters (measured values), the former is consistently lower (Table 8).

**Table 8:** Energy and water consumption results in Paricarana campus buildings.

	Calculated Value	Measured Value	Difference (%)
Energy (MWh/year)	5002	5205	4.06%
Water (m³/year)	3994	4389	9.89%

This result can be explained by the fact that only the most representative CE were accounted for on the calculated values. Water and energy consumption measured in university takes many forms. Factors affecting consumption, for example, of water include swimming pools or large garden areas (Cheng and Hong, 2004). Thus, the calibration of the system was done by adding to the consumption of buildings a new consumption element called other consumption (OC). This element was quantified considering the differences between calculated and measured values.

### 5.4 End-use consumption

The water and energy consumption was detailed only for the most significant end uses. Total energy consumption was divided into four components: i) lighting; ii) air conditioning; iii) electronic devices; and iv) other energy consumption (OEC). Total water consumption was divided into three components: i) personal use (WCP); ii) cleaning (WCC); and iii) other water consumption (OWC).

**Figure 7:**
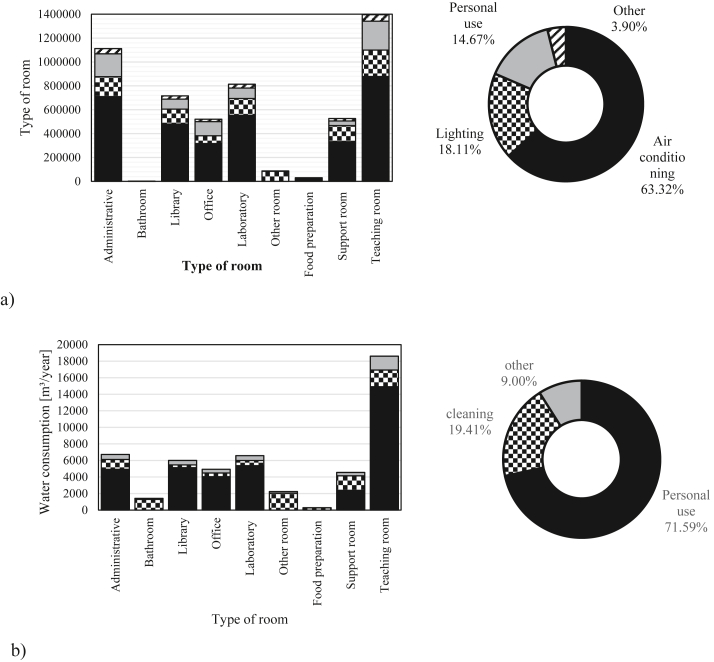
Energy consumption by type of rooms and end-use (a) and water consumption type of rooms and end-use (b).

Regarding energy consumption, air conditioning accounts for 63.32% of consumption, followed by lighting with 18.11% and personal use with 14.67% (Figure 7 a). Most of the energy consumption takes place in classrooms and administrative rooms, representing 48% of the total. As for water consumption, personal use represents 72% and cleaning activities 19% (Figure 7 b). The presence of teaching rooms in buildings increases water consumption, as it is the room that concentrates most of the population (39%). The food preparation room was the one with the lowest consumption, since the water consumed for preparing food was not taken into account.

The energy and water use intensity per end-use of buildings of the Paricarana campus is shown in Table 9. Regarding energy consumption, the libraries, administrative areas and offices are the biggest consumers, because the rooms are refrigerated and have the longest hours of operation. Bathrooms and other rooms have the lowest consumption because they are rooms without refrigeration. In general, air conditioners (ECAC) have the highest values of energy use intensity. For water consumption, the biggest values of the water use intensity are for the library, office and teaching rooms, as these are the rooms with the highest occupation rates. Personal use in general has the biggest indicator. Thus the energy consumption is more related to the time of use of the rooms, while the water consumption is driven by the occupancy of the rooms.

**Table 9:** Consumption intensity per end-use by type of room.

Code	AA	BA	LIA	OA	LA	OTA	FPA	SA	TA
**Energy (kWh/m**^**2**^**/year)**									
ECAC	84.30	0.00	199.35	99.81	65.82	0.00	15.90	26.60	55.93
ECL	19.81	0.38	51.81	20.41	16.67	5.80	3.18	10.85	14.02
ECE	22.80	0.00	34.68	37.40	10.41	0.00	0.00	3.12	15.44
COE	5.15	0.02	11.60	6.40	3.77	0.24	0.77	1.65	3.47
TEC	132.07	0.40	297.44	164.02	96.66	6.03	19.86	42.22	88.85
**Water (m³/m**^**2**^**/year)**									
WCP	0.59	0.00	2.13	1.27	0.64	0.00	0.00	0.19	0.95
WCC	0.14	0.36	0.14	0.14	0.07	0.14	0.18	0.14	0.13
OWC	0.07	0.04	0.22	0.14	0.07	0.01	0.02	0.03	0.11
TWC	0.80	0.40	2.50	1.55	0.78	0.16	0.20	0.37	1.18

## 6 Discussion

It is difficult to compare consumption between different universities for several reasons. Universities, in general, have buildings with different characteristics, due to the location, activities developed, nature, among other factors. No published data was found in the literature with buildings similar to the UFRR buildings, both in climatic conditions and in physical characteristics. However, the values presented in this study are within the expected ranges when compared to the other studies presented in the Table 10.

**Table 10:** Comparison of water and energy consumption in the current study with other authors.

Reference	Location/climate	Type variable	(kWh/m^2^/year)	(kWh/hab/year)	(m³/m^2^/year)	(m³/hab/year)
This study	**Brazil/Aw**	**Room type**	**0.4–297.44**	**963.54**	**0.2–2.5**	**15.12**
(Zhou et al., 2013)	Chine/Cfa	Disciplines/nature	37–10	400–1480	0.8–3.5	38–176
(Abdelalim et al., 2015)	Canada/ET	Building type	40–500	1000–104.340	0–16	0–110
(Escobedo et al., 2014)	Mexico/Cwa	Room type	18.15–95.83	-	-	-
(Malandrakis et al., 2017)	Accidental Macedonia/Dfb	General		150–240		1
(Bonnet et al., 2002)	France/Cfb	Room type	25–123	-	0.17–3.99	-
(Amber et al., 2017)	England/Cfb	Building type	155–426			
(Hong et al., 2011)	Korea/Dfa ​unive	General	211–252.63	3888.88–4444.44		
(Rewthong et al., 2015)	Thailand/Cfa	General	79.98–87.57			
(Mccusker, 2013)	USA/Cfa	Building type	252.3–567.7	1758.5–7913.24		
(Batlle et al., 2020)	Brazil/Cfa	General	243–554			
(Marinho et al., 2019)	Brazil/Af	General				9.782
(Wichowski et al., 2019)	Poland/Cfb	General				6.5–8.95
(L. Li et al., 2017)	Chine/Cfa	General		707.27–1415.70		
(Qu et al., 2015)	Chine/Cfa	General		1 097		94
(Ó Gallachóir et al., 2007)	Ireland/Cfb	General	145–187	1480–1590		
(Bourdeau et al., 2018)	France/Cfb	Building type	52.40–362.7 (pe)			
(Ward et al., 2008)	England/Cfb	Building type	240–339	2492–9187		
(Khoshbakht et al., 2018)	Australia/Cfb	Building type	136–164			
(Wang, 2016)	Taiwan/Am	Nature	63.8–90.4	927–2889		

Studies on the relationship between the use of energy and the use of rooms in higher education buildings have shown that the energy consumption of buildings has a strong relationship with the activities developed in the rooms of the buildings (e.g., Gui et al., 2020).

The rooms with the highest intensity of energy and water use at Campus Paricarana are the libraries (297.44 kWh/m^2^/year and 2.5 m³/m^2^/year), which is in accordance with the study by Liu and Ren (2020).

After the library, the offices and administrative areas have the highest levels of energy use intensities (162.02 kWh/m^2^/year and 132.07 kWh/m^2^/year), being higher than those found in some studies (Bonnet et al., 2002; Escobedo et al., 2014; Gui et al., 2020). This may be justified by the specificities of the campus rooms, which are cooled throughout all the periods of use.

The average intensity of energy consumption associated to rooms’ lighting is 16 kWh/m^2^/year, which is in line with studies by Escobedo et al. (2014).

The consumption and end use of water in universities are less addressed in the literature than energy consumption. Regarding end uses, water consumption for personal use on campus corresponds to 72% of the total, with toilets representing 58% of that consumption, in line with those reported by Meireles et al. (2014) and Marinoski and Ghisi (2008).

It appears that the greatest difficulty in finding reference values for universities lies in the fact that consumption in these institutions is hard to categorize and to define patterns. Although different activities are carried out in universities, in general, they have similarities between the types of existing rooms, which are mostly classrooms, libraries, laboratories, among others. In this context, consumption indicators by type of class, considering the main elements of consumption, provide values that can be easily compared. In principle, the water and energy intensity consumption in classrooms of universities in similar climatic conditions, should have some similarities. High consumption levels will tend to indicate poor performance, while low indicate good performance in relative terms.

In this context, the main advantage of the presented methodology is to provide a characterization of university rooms, which makes it possible to formulate indicators that can be easily compared between different institutions. This allows to quantify the degree of efficiency of different universities in terms of energy and water consumption. The absence of data makes it difficult to evaluate the use of these resources in the universities.

## 7 Conclusion

This paper presents a methodology to estimate water and energy consumption patterns per end-use in buildings by modeling its drivers, namely the function and features of the building rooms and the characteristics of their main users. By enabling the estimate of water and energy consumption patterns, the proposed methodology is useful for managers and decision-makers of buildings because it provides the basis for assessing the performance of potential water and energy efficiency measures. Also, it enables benchmarking the energy and water performance of between buildings.

The methodology is applied to the Paricana Campus of the Federal University of Roraima, in Brazil. The buildings on the Paricarana Campus do not have individual water or energy measurements, so the mixed method was used to estimate the consumption of each building per end-use. The water and energy consumptions per end-use in each building were estimated indirectly and then calibrated based on sectoral measurements. In a first stage, the results on the total consumption per sector obtained with the proposed model were slightly lower than the measured ones, 4.06% for energy and 9.89% for water. Therefore, in a second stage, a calibration was introduced by adding a new consumption element to the system, with values corresponding to the observed differences.

The methodology uses buildings composition and main activities in each roomto estimate the consumptions indirectly. The use of both variables is important, as proved with the case study. The university campus under study has mostly teaching rooms (22%) and other rooms (21%), but the energy and water consumption varies a lot in each one of these roomtypes: between 88.85 kWh/m^2^/year and 6.03 kWh/m^2^/year for energy consumption and between 1.18 m³/m^2^/year and 0.16 m³/m^2^/year for water consumption. Therefore, also the main activities developed in each type of roomneed to be taken into account when estimating the consumption patterns.

For the Paricarana Campus, most energy consumption is associated with the cooling of rooms, representing 63%, followed by lighting and personal use, which represent 18% and 15% respectively. As for water, the largest portion is represented by personal use, corresponding to 72%, 59% of each for toilets and 23% for washbasins. It appears that the energy consumption is more linked to the time of use of the rooms, since all rooms in which permanent activities are developed are refrigerated, regardless of the occupancy rates. With regard to water consumption, personal use is the most relevant part, so consumption increases with the increasing population density of the building. In this sense, the building that presented the highest consumption was the Central Library because it had longer operating times and higher occupancy rates.

The methodology proposed to estimate consumption in buildings proved to be effective, especially in buildings with multiple activities. It also presents itself as an adequate solution for campus where the consumption measurements are not individualized by building and presents a structure that allows the generation of indicators that can be used to estimate and compare consumption between different buildings. Additionally, all these features facilitate the creation of databases supporting the assessment of the energy and water efficiency of buildings.

## Declarations

### Author contribution statement

Alissandra Pessoa Almeida: Conceived and designed the experiments; Performed the experiments; Analyzed and interpreted the data; Wrote the paper.

Vitor Sousa: Conceived and designed the experiments; Analyzed and interpreted the data; Wrote the paper.

Cristina Matos Silva: Conceived and designed the experiments; Wrote the paper.

### Funding statement

This research did not receive any specific grant from funding agencies in the public, commercial, or not-for-profit sectors.

### Data availability statement

The authors do not have permission to share data.

### Declaration of interests statement

The authors declare no conflict of interest.

### Additional information

No additional information is available for this paper.
